# Cytokine and Phenotypic Cell Profiles of *Leishmania infantum* Infection in the Dog

**DOI:** 10.1155/2012/541571

**Published:** 2011-08-09

**Authors:** Carla Maia, Lenea Campino

**Affiliations:** ^1^Unidade de Parasitologia Médica, Instituto de Higiene e Medicina Tropical (IHMT), Universidade Nova de Lisboa (UNL), Rua da Junqueira 100, 1349-008 Lisboa, Portugal; ^2^Centro Malaria e Doenças Tropicais, IHMT/UNL, Rua da Junqueira 100, 1349-008 Lisboa, Portugal; ^3^Department of Parasitology, Faculty of Sciences, Charles University, Prague, Czech Republic; ^4^Departamento de Ciências Biomédicas e Medicina, Universidade do Algarve, Campus Gambelas, 8005-139 Faro, Portugal

## Abstract

Leishmaniasis has reemerged in recent years showing a wider geographic distribution and increased global incidence of human and canine disease than previously known. Dogs are the main domestic/peridomestic reservoir hosts of zoonotic visceral leishmaniasis caused by *Leishmania infantum*. Since the evolution of leishmaniasis and clinical appearance is a consequence of complex interactions between the parasite and host immune response, a profound knowledge about the immune profile developed in dog's infection is crucial for vaccine and immunomodulatory therapy design. The main goal of this paper is to compile the recent advances made on cytokine and phenotypic cell profiles in different tissues and organs of dogs infected with *L. infantum*. This paper also stressed that the knowledge of the immune responses developed, namely, in liver, lymph node, and spleen is very limited. All data emphasizes that more research on canine leishmaniasis is necessary for the development of new and efficacious tools to control zoonotic leishmaniasis.

## 1. Introduction

Canine leishmaniasis (CanL) caused by *Leishmania infantum* (syn. *L. chagasi*, in Latin America), which is transmitted by the bite of phlebotomine sand flies, is endemic and affects millions of dogs in the Mediterranean basin, China, and Latin America and is an emergent disease in North America. Dogs are described as the best experimental animal model for visceral leishmaniasis caused by *L. infantum*, because many of the clinicopathological signs and immune responses observed in experimental CanL are similar to those observed on natural canine and human* Leishmania* infection [1, 2].It has been claimed that dogs never achieve parasitological cure, and the widespread use of the available anti-*Leishmania* drugs for both canine and human treatment may contribute to parasite drug resistance. Therefore, an efficacious CanL vaccine able to block parasite transmission would be the best strategy to control the spread of the disease among other dogs and an essential part for the control of human zoonotic leishmaniasis [3]. The development of efficient immunoprophylactic molecules to maintain long-term immunity and to promote leishmaniases control relies on the identification and the characterization of the immune events associated with disease progression.

Clinical appearance and evolution of leishmaniasis is a consequence of complex interactions between the parasite and the genetic and immunological background of the host. It is widely accepted that in susceptible animals the progression of infection to active disease is characterized by a marked humoral response, a cellular immune depression against the parasite, and the appearance of a full array of clinical signs. On the other hand, resistant dogs lack clinical signs, develop low levels of anti-*Leishmania* antibodies and parasite load, and develop a strong *in vitro *lymphocyte proliferative response and a positive delayed-type hypersensitive response to leishmanial antigens in the skin [4–6]. Nevertheless, it is important to keep in mind that animals considered resistant could be in an earlier stage of disease prior to developing signs of susceptibility [7].

Determining the role of T-helper-1 (Th1) and Th2 lymphocyte subpopulations in different tissues and organs of infected dogs is crucial to understand the immune mechanisms induced by infection. Previous studies described that cellular immune response in CanL was associated with activation of Th1 cells producing interferon-gamma (IFN-*γ*), interleukin-2 (IL-2), and tumour necrosis factor alpha (TNF-*α*) while cytokine pattern of active disease was characterized by a mixed Th1/Th2 response [5]. However, most of these works were made on peripheral blood (PB). Furthermore, other studies showed that the immune response to the parasite is not identical in whole host system but instead organ-specific [8]. In fact, a Th1, Th2, or mixed Th1/Th2 immune responses were observed in different organs of dogs infected with *L. infantum* and correlated with the presence or the absence of clinical signs and local parasite load [7, 9–12]. The organ-specific and mixed Th1/Th2 immune responses were also verified in leishmaniasis murine model [13, 14]. 

The challenges regarding local and systemic immune responses to the parasites need to be answered to achieve the development of efficacious strategies to control canine and human visceral leishmaniasis. The aim of this paper was to describe the recent studies about the cytokine and cell population profiles developed in different target organs/tissues, namely, bone marrow, lymph node, liver, peripheral blood, skin, and spleen developed by the dog to *L. infantum* infection.

## 2. Tissue and Organ Immune Responses in Canine Leishmaniasis

### 2.1. Peripheral Blood

IFN-*γ* expression or production by nonstimulated peripheral blood mononuclear cell (PBMC) lymphocytes or stimulated with soluble *Leishmania *antigen (SLA) from infected dogs has been correlated with disease resistance/asymptomatic status in both nonvaccinated and vaccinated animals [15, 16] as well as in dogs challenged with uninfected or infected colony reared *Lutzomyia longipalpis* [17]. Taking into account the results obtained in those works, the no expression of IFN-*γ* by PB (Table 1) could have been related with the persistence of infection in an experimental canine study performed in our laboratory [6]. On the other hand, Travi et al. [11] observed that the PMBC stimulated with SLA from 67% of symptomatic dogs experimentally infected with promastigotes isolated from the vector *Lutzomyia longipalpis* produced high levels of IFN-*γ* at the early stages of infection, and the proportion of individuals producing this cytokine increased over time, indicating that IFN-*γ* production and expression was not sufficient to prevent disease and, consequently, was not a good marker of resistance. On opposite, Carrillo et al. [16] found a depleted expression of IFN-*γ* in response to SLA in symptomatic experimentally infected dogs; according to Carrillo and Moreno [5], the low expression of this cytokine might have been associated with a diminished CD4+ T lymphocyte subset population. Several studies analysing lymphocyte subtypes by flow cytometry have pointed out that this PB population in sick dogs is decreased but returns to normal values after treatment [18–20], others observed an increased number of CD4+ cells in dogs with low parasitism [21]. Furthermore, others found out that the number of CD4+ T-cells in PB was similar in dogs with leishmaniasis and in healthy dogs and that there was no correlation between the clinical status or response to therapy and CD4+ counts [22, 23]. The contradictory results obtained highlights the complexity of the immune response mounted in response to *L. infantum* infection and that this lymphocyte subtype cannot be used alone as a prognosis marker. Reduction in CD3+ and increased CD5+ lymphocyte subpopulations in PB has been described in symptomatic dogs [18, 21, 23] while high CD8+ numbers were detected in animals with low parasitism [19, 21]. It was observed that a decreased CD21+ B cells, CD14+ monocytes, and class II molecules of the major histocompatibility complex (MHC-II) in symptomatic dogs were related with selective migration into lymphoid organs and lower ability for antigen presentation [21]. The contradictory results obtained were probably due to the comparison of the blood leukocyte subpopulations and parasite loads in different compartments. Moreover, the differences obtained between studies could also be related with the classification of the animals not only with the absence or presence of clinical signs but also with the development of specific anti-*Leishmania *antibodies or the detection of parasite since each methodology presents different sensitivity and specificity. 

Regarding the expression and/or production of other cytokines in peripheral blood, results are also discrepant. While for some authors interleukin-6 (IL-6) [24] and IL-18 [25] are markers of active disease or asymptomatic infection; respectively, for others IL-6 [26] and IL-18 [15, 16] have no determinant role. Similar results were obtained for IL4 and IL10, where their expression was only observed in mitogen-stimulated PBMC from symptomatic dogs [27] while others detected it in animals presenting clinical signs or not [15, 16]. From these studies, IL-4 did not seem to contribute to canine susceptibility to infection. On the other hand, the increase of IL-10 production by PBMC stimulated with SLA along with increase blood parasite burden has recently pointed out to be predictive of the evolution of canine infection [7]. Others have verified the association between IL-10 and active visceral leishmaniasis in humans [28]. Nonetheless, the spectrum of cytokines and the immunophenotypic of cells cannot be considered good markers to predict the evolution of infection since PB is not the tissue of election for parasite multiplication and persistence.

### 2.2. Skin

Skin is essential for the transmission of *Leishmania* since it is the tissue where an infected sand fly inoculates parasites into the vertebrate and the first barrier of the immune system. In the study performed by Solano-Gallego et al. [29], muzzle skin of asymptomatic dogs had no histological demonstration of lesions neither amastigotes, but parasitesDNA was detected. According to these authors, asymptomatic dogs found positive to *Leishmania* parasites by PCR do not play a significant role in the infection of phlebotomine sand flies. However, in a recent work performed by Madeira et al. [30], *L. chagasi* was isolated from intact skin from different body regions of 292 out of 394 seropositive dogs despite that only 21.9% of them were recognised as symptomatic. Furthermore, Guarga et al. [31] through xenodiagnosis concluded that asymptomatic dogs were infectious to the sand fly vectors. All these data suggest that parasites have large distribution in the skin of infected dogs in spite of remaining asymptomatic for prolonged time and for that reason not submitted to any control measures. The presence of parasites in these animals highlights their importance as reservoir hosts. Thus, from an epidemiological point of view, it would be important to correlate the local immune response of skin to the presence of the parasite as well as with the infectiousness to competent vectors and therefore, allow the development of tools for blocking transmission.

A variety of cells, such as intraepithelial T lymphocytes and Langerhans cells, are present in the skin and are capable to generate local immune reactions. Branchelente et al. [9] demonstrated that the local immune response in lesional skin of naturally infected dogs included IFN-*γ*, TNF-*α*, and IL-4 expression. The increased expression of IL-4 was associated with severe clinical signs and a high parasite burden in the skin biopsies. More recently, Menezes-Souza et al. [12] analysed the expression of proinflammatory, anti-inflamatory, and immunoregulatory cytokines as well as the levels of transcription factors T-bet (associated with Th1 immune response), GATA-3 (associated with Th2 immune response), and FOXP3 (involved in the regulation of cytokine gene transcription) in the skin without lesion of dogs naturally infected. A mixed Th1/Th2 cytokine profile was observed in asymptomatic dogs. Additionally, low levels of transcription factors GATA-3 and FOXP3 were also correlated with the absence of clinical signs. The data obtained indicate that in asymptomatic infection or in cases with lower skin parasitism, a mixed inflammatory and regulatory immune response profile may be of major relevance for both the maintenance of the clinical status of the dogs as well as for parasite replication at low levels. 

Fondevila et al. [32] and Papadogiannakis et al. [33] investigated the cellular immunophenotyping and the number of amastigotes in the epidermis and dermis in dogs with patent leishmaniasis. For these authors, alopecic and sebaceous adenitis were associated with an effective local immune response characterized by the activation of epidermal Langerhans cells, upregulation of MHC-II molecules on keratinocytes, dermal infiltration by CD8+ and a lower number of CD4+ cells, presence of CD21+ cells, and a relatively low parasite burden. On the other hand, dogs with generalized nodular disease mounted an impaired immune response characterized by a low epidermal expression of MHC-II molecules, small numbers of T cells in the dermal infiltrate, and a high parasite burden [32]. Taking into account data obtained in several studies, it would be important to determine the immunophenotype associated with the absence of parasites in the skin.

### 2.3. Lymph Nodes

Lymph nodes (LN) are widely accepted to be the first relevant lymphoid tissues affected after dissemination of the parasite from skin macrophages; thus, the evaluation of their immune response to* Leishmania* might help in determining the infection outcome. However, the cellular immune responses developed in LN to *L. infantum* are scarce. Giunchetti et al. [34] analysed the immunophenotypic profile in popliteal LN from naturally infected dogs and its relation with parasite burden in this organ and skin. A significant increased number of T lymphocytes, particularly CD8+ cells, in addition to decreased levels of CD21+ B cells and upregulation of MHC-II molecules were the major LN immunophenotypic changes observed. Interestingly, the highest number of CD8+ T cells was observed in animals harbouring the highest skin parasitism. According to the authors, LN CD8+ T cells may present a distinct activation status during CanL, probably associated with immunomodulatory or suppressor cell activity. In fact, the immunomodulatory effect of these cells was recently observed by Alexandre-Pires et al. [23] where that CD8+ subpopulation in LN from treated dogs was significantly lower than in asymptomatic dogs. Moreover, CD4+ T-cell subset in LN from both asymptomatic and treated dogs was significantly higher than that in noninfected dogs. Together, these findings suggested that lymphocyte activation in the LNs with the expansion of CD4+ subpopulation may favour the control of *Leishmania* infection through a local reduction of parasite replication and/or parasite clearance while an increase of the number of CD8+ cells seem to be related with parasite persistence and immunomodulatory cell activity.

Regarding cytokine profile, Alves et al. [35] evaluated its relation with parasite burden in prescapular LN from naturally infected dogs and observed that the balance of expression of IFN-*γ*, TNF-*α*, IL-10, and transforming growth factor-beta (TGF-*β*) determines parasite load and clinical expression. LN from asymptomatic dogs had higher expression of proinflamatory cytokines and lower number of parasites indicating that IFN-*γ* and TNF-*α* could play a role in protection against disease while LN from symptomatic dogs expressed more anti-inflamatory cytokines suggesting a role for IL-10 and TGF-*β* in disease progression. This event is in agreement with the cytokine profile observed by us in popliteal LN from dogs experimentally infected where the balance between the percentage of IL-10 and TNF-*α* expression (Table 1) could have been the responsible not only for a lower parasite load in this tissue than in others (skin, hepatic, and splenic), but also for the absence of lymphadenomegaly or other clinical signs at the end of the study in most of the infected dogs (only one dog had popliteal adenomegaly). 

All these data highlight that more studies focused on specific immunological events in LN, namely, on cytokine profile and CD8+ T and CD4+ subpopulations, should be performed in order to determine if triggering an effective immune response in this lymphoid tissue could avoid an intense multiplication and consequent dissemination of the parasite to other organs [35].

### 2.4. Liver

One of the most relevant organs involved in the parasite-host interface during *L. infantum *infection is the hepatic compartment. However, and as far as we are aware, only one study quantified the cytokine production by the liver of dogs infected and observed that the production of IFN-*γ*, IL-10, and TGF-*β*1 was higher in those with no clinical signs [36]. Our results on experimental CanL correlated these findings as liver cells of the infected animals also expressed those cytokines as well as iNOS (Table 1). On the other hand, there was no expression of TNF-*α* and IL-4. The presence of interlobular granulomas of variable severity in infected dogs could be a reaction of the organism to parasitism in an attempt to control the multiplication of the parasites [37]. The presence of hepatic granulomas was also correlated with subclinical human VL [38]. In addition, Stanley and Engwerda [39] suggested that apart from IFN-*γ* responsible for the generation of leishmanicidal mechanisms, TNF-*α* is also involved in hepatic granuloma formation and contributes to the resolution of local infection in the murine model. Thus, the no expression of TNF-*α* by the hepatic cells of our experimentally infected dogs could have been associated with the high parasitism observed [2]. 

As mentioned above, data regarding cytokine profile are also quite limited highlighting the necessity to perform more studies in order to improve our knowledge concerning the immune response developed in liver during visceral* Leishmania* infection.

### 2.5. Spleen

CanL is associated with splenic architecture disruption, which is characterized by disorganization of normal lymphoid tissue, loss of normal spleen leukocyte diversity via replacement of leukocytes by plasma cells, and eventual atrophy of the lymphoid tissue [40]. Thus, whilst the spleen is responsible for the major immune response in leishmaniasis, the present knowledge of the cytokines and leukocytes that participate in its immune response is very limited since few studies were performed. RNA expression levels of a wide range of cytokines (IFN-*γ*, TNF-*α*, IL-4, IL-5, IL-10, IL-12, IL-18, and TGF-*β*), transcription factors (T-bet and GATA3), and chemokines (IP-10, RANTES, MIP-1*α*, MCP-1) were evaluated in the spleen from naturally and experimentally infected dogs [10, 41]. A positive correlation between the expression of IL-10 by splenocytes with both increased parasite load and progression of the disease was observed in naturally infected dogs [41]. According to Santana et al. [42], the production of IL-10 within splenic granulomas may provide immunological conditions for the survival and growth of the parasite. On the opposite, Strauss-Ayali et al. [10] did not find any change in the expression of IL-10 by splenocytes throughout experimental infection with 8.6 × 10^8^ *L. infantum *amastigotes, even in animals with a high parasite load. Similar result was obtained by Corrêa et al. [36] where no differences were found in the production of IL-10 by spleen extracts between symptomatic and asymptomatic naturally infected dogs. On the other hand, Strauss-Ayali et al. [10] suggested that the early increase of IL-4 might have a role in the persistence of parasites in the presence of high IFN-*γ* expression. An association between high levels of IFN-*γ* and chemokines expression and splenic parasitism with a worst disease prognostic was also observed [10, 36, 41]. Interestingly, in our experimental infected dogs, IFN-*γ* was only expressed by the tissues with high parasite load, namely, spleen, bone marrow, and liver (Table 1), suggesting that the presence of this cytokine is not synonymous of parasite clearance. One possible explanation for the association between IFN-*γ* presence and the high parasite load could be that new parasite generations are constantly being seeded from other infected tissues stimulating its expression [10]. Furthermore, the levels of IFN-*γ* and chemokines expression significantly decreased after treatment, reflecting a reduced recruitment of immune cells into the spleen due to the minimal amount of parasites remaining in the organ. It is important to mention that at this moment the source of such IFN-*γ* is not known, and probably it is not produced by T cells, since symptomatic dogs show T-cell depletion in the spleen and a specific immunosuppression against the parasite.

The elevated levels of the tested chemokines observed by Strauss-Ayali et al. [10] were suggestive of an accumulation of infiltrating monocytes attracted by MIP1-*α* and MCP-1, as well as of CD4+Th1 and CD8+ cells which could have been recruited by IP-10. In agreement with these data, Guerra et al. [19] observed an increased frequency of CD8+ T cells in the spleen with low parasite load.

Nevertheless, in the few studies performed up to now, none of the Th2 [36], Th1/Treg [41], or Th1/Th2 [10] cytokine immune responses neither the chemokine nor phenotypic cell profiles obtained were able to eliminate the parasite locally.

### 2.6. Bone Marrow

Progression of *Leishmania* infection has been related with a granulomatous inflammation in bone marrow accompanied by an increased percentage of lymphocytes and plasma cells, erythroid and megakaryocytic hypoplasia, and/or dysplasia and erythrophagocytosis [4]. According to Manzillo et al. [43], megakaryocytic and erythroid dysplasia were probably related to an increased number of bone marrow macrophages producing high levels of TNF-*α* and IFN-*γ*. In agreement with this hypothesis, Quinnell et al. [44] observed an increased accumulation of these cytokines in bone marrow of naturally infected dogs with and without clinical signs. These authors also detected a significant positive correlation between disease severity and IL-4. However, this cytokine was not expressed by the bone marrow cells of dogs experimentally infected six months after infection (Table 1). Instead, iNOS and a mixed pattern of proinflamatory (TNF-*α*) and regulatory (TGF-*β* and IL-10) cytokines were detected in those asymptomatic dogs. Thus, one of the reasons for the asymptomatic evolution of the infection observed in those dogs despite the high parasite load in bone marrow, liver, and spleen could have been related with the no expression IL-4 by these organs. 

In a study on bone marrow leukocyte subpopulations in naturally infected dogs with and without clinical signs of CanL and after being treated for leishmaniasis, Alexandre-Pires et al. [23] observed that symptomatic and asymptomatic animals exhibited a significant increase of MHC-II expression in bone marrow lymphocytes probably reflecting the presentation of* Leishmania* antigens. Moreover, treated animals also showed increased expression of MHC-II monocytes pointing out to elevated levels of antigenic presentation activity, possibly due to the availability of parasite antigens as a consequence of treatment. No differences in CD8+ and CD4+ T-cell populations were observed between the three studied groups allowing hypothesizing that the control of infection in the bone marrow is not related with the expansion of these cells.

## 3. Conclusions

The cytokines and phenotypic cell profiles that participate in immune responses in different compartments where the parasite replicates seem to have variable effects on local parasite control, highlighting the complexity of the cellular immune response developed by the dog to *L. infantum* infection. Moreover, these studies have disclosed interesting facets of the immune response, even contradicting some dogmas such as the role of IFN-*γ* in parasite clearance. Furthermore, this paper also stressed that the knowledge of the immune responses in some lymphoid compartments (liver, lymph node, and spleen) is very limited. 

Since the presence and intensity of parasites in blood in CanL are normally low and transient, the generation of immunological data in this tissue might not be an accurate reflection of immune responses that occur in the body compartments where a high parasitism is normally observed. On the other hand, could the immune response in PB be the result of the immune responses developed in the different organs and thus be used as a prognosis marker? Or could local immune response developed in spleen, the major lymphoid organ involved in parasite-host interaction, be used to predict the evolution of infection? In fact, here, the invasive method of sampling must be considered.

Future integrated studies are needed in order to clarify the association between the trafficking of various cell subpopulations, cytokine, and chemokine gradients in association with the immune responses and parasite loads in the different visceral and peripheral tissues developed at the same time and in the same animal in order to improve the knowledge of local and systemic immune responses in CanL. The effective immune response able to control the parasite must be evaluated in animals with no clinical signs and with very low parasite burden since only these animals can be considered immune competent/resistant to clinical disease. In addition, the identification of specific cell subpopulations that are involved in disease control in different organs will allow control strategies namely, the development of efficacious therapeutic and prophylactic tools.

##  Conflict of Interests

All authors declare that they have no conflict of interests concerning the work reported in this paper.

##  Ethical Guidelines

The study on *Leishmania infantum* experimental infected dogs followed the International Guiding Principles for Biomedical Research Involving Animals and the guidelines of the Portuguese Legislation (Lei n°92/95, 12.9) and was approved by the Ethics Committee of the Instituto de Higiene e Medicina Tropical, Universidade Nova de Lisboa.

## Figures and Tables

**Table 1:** Frequencies of cytokine and iNOS expressions determined by reverse transcriptase PCR in 12 asymptomatic dogs (with high parasite load in viscera) infected with *L. infantum *amastigotes, six months postinfection.

Tissue/organ	INF-*γ* (%)	TGF-*β* (%)	TNF-*α* (%)	IL-10 (%)	iNOS (%)
Peripheral blood	0	91.66	66.66	0	0
Lymph node	0	36.36	54.54	81.81	36.36
Liver	9	18.16	0	18.19	18.19
spleen	11.11	11.11	0	33.33	33.33
Bone marrow	25	66.66	41.66	100	75

INF-*γ*: interferon-gamma; TGF-*β*: transforming growth factor-beta; TNF-*α*: tumour necrosis factor-alpha; IL-10: interleukin-10; iNOS: inducible nitric oxide synthetase.
